# Novel Human Parvovirus 4 Genotype 3 in Infants, Ghana

**DOI:** 10.3201/eid1607.100025

**Published:** 2010-07

**Authors:** Marcus Panning, Robin Kobbe, Silke Vollbach, Jan Felix Drexler, Samuel Adjei, Ohene Adjei, Christian Drosten, Jürgen May, Anna Maria Eis-Hübinger

**Affiliations:** Author affiliations: Institute of Virology, Bonn, Germany (M. Panning, S. Vollbach, J.F. Drexler, C. Drosten, A.M. Eis-Hübinger);; Bernhard Nocht Institute for Tropical Medicine, Hamburg, Germany (R. Kobbe, J. May);; Ministry of Health/Ghana Health Service, Agona, Ghana (S. Adjei);; Kumasi Centre for Collaborative Research in Tropical Medicine, Kumasi, Ghana (O. Adjei)

**Keywords:** PARV4, genotype 3, infants, viruses, Ghana, dispatch

## Abstract

Human parvovirus 4 has been considered to be transmitted only parenterally. However, after novel genotype 3 of parvovirus 4 was found in 2 patients with no parenteral risks, we tested infants in Ghana. A viremia rate of 8.6% over 2 years indicates that this infection is common in children in Africa.

In 2005, a novel human parvovirus, termed parvovirus 4 (PARV4), was identified in a plasma sample from a patient with symptoms resembling those of acute HIV infection (*1*). In 2006, a related virus was discovered in plasma pools for manufacture of plasma-derived medical products; the virus was initially called PARV5 and is now called PARV4 genotype 2 (*2*). Phylogenetic analysis suggested that PARV4 formed a separate novel genus within the subfamily *Parvovirinae*.

Initial PCR analyses of blood and autopsy specimens from adults suggested that PARV4 infection was restricted to persons at risk for parenteral infection with viruses such as hepatitis C virus (HCV) or HIV (e.g., injection drug users) (*3**–**5*). A recent serologic study identified high frequencies of immunoglobulin G against PARV4 in injection drug users who were co-infected with HCV and HIV and in persons with hemophilia who had been exposed to non–virus-inactivated clotting factor concentrates (*6*). Absence of serologic reactivity in adults without parenteral risk factors supported an association between PARV4 and blood-borne transmission.

Only limited genetic diversity has been found among PARV4 sequences, particularly among genotype 1 viruses, suggesting recent emergence and spread among parenterally exposed persons in Europe and the United States (*5*). Because persons with PARV4 genotype 1 DNA in their tissues have been substantially younger than those with genotype 2, a recent shift in prevalence of the 2 genotypes has been suggested; genotype 1 currently predominates (*4*). An even more pronounced shift was found for parvovirus B19 in that nearly complete cessation of genotype 2 and replacement by genotype 1 occurred in the 1960s in western countries (*7*). Furthermore, molecular clock analysis indicated that B19 genotype 3, which is endemic to West Africa and rarely detected outside Africa, may be more ancient than genotypes 1 and 2 (*8*).

Recently, a novel PARV4 variant, termed genotype 3, was identified in tissue samples of 2 adults from Nigeria and the Democratic Republic of the Congo (*9*). Each patient had signs of AIDS but was antibody negative for HCV and had no evidence of parenteral exposure. The absence of parenteral risks raises the possibility of alternative routes of transmission that might affect the general, nonparenterally exposed, population (*9*). Proving nonparenteral transmission would suggest more widespread distribution of PARV4 in humans than previously expected and occurrence of virus in additional population subsets. We therefore studied the occurrence of PARV4 in infants in Ghana.

## The Study

We analyzed 279 anonymous blood samples that had been collected during a trial of intermittent preventive malaria treatment for infants from January 2004 through September 2005 (*10*). Samples came from infants from 9 villages in the rural Afigya Sekyere district, Ashanti region, Ghana, where estimated prevalences of HIV-1 and HCV in adults were <3% (*10*) and 2.5% (*11*), respectively. For storage under tropical conditions, an equal volume of whole blood in EDTA was supplemented with buffer AS1 (QIAGEN, Hilden, Germany). DNA isolation was conducted on a BioRobot M48 workstation (QIAGEN), using the MagAttract M48 DNA Mini Kit (QIAGEN) as recommended (sample input volume 200 µL, elution volume 200 µL).

All samples were first tested by a previously described real-time PCR designed to detect established PARV4 genotypes 1 and 2 (*12*). When initial sequencing identified novel PARV4 genotype 3, a specific real-time PCR for this genotype was developed by using primers 5′-ACCAAGGACACCAGACAGTCTT-3′ and 5′-ACGTGTTCAGACCAAAAGGAT-3′ and probe 5′-FAM-CCAGCTCCATACCTTTCAGCAGTTGC-BHQ1-3′. All samples were retested by using a plasmid-based standard derived from the real-time PCR amplicon of sample Ghana19 for absolute quantification of genome copy numbers. The lower limit of detection of this assay was 10 plasmid copies/reaction. Procedures to prevent PCR contamination were strictly adhered to, and negative controls were used throughout.

In total, 24 (8.6%) of 279 samples were positive for PARV4 genotype 3 DNA. At the time of blood collection, no infant had signs of acute infection (fever, rash, myalgia). Positive samples were found for infants in 7 of 9 studied villages, indicating widespread prevalence. To test whether socioeconomic factors might influence prevalence, we conducted a χ^2^ test. A significantly lower relative risk of acquiring PARV4 infection was found for children who had access to a kitchen (indicative of higher level of hygiene within household) and who did not live close to a river (Table).

**Table:** Socioeconomic risk factors associated with PARV4 genotype 3 in whole blood from infants, Ghana, January 2004–September 2005*

Factor†	No. tested‡	PARV4 viremia until month 24		
		No. (%) positive	RR (95% CI)	p value§
Kitchen available				
No	137	15 (11.0)	1	
Yes	126	5 (4.0)	0.36 (0.14–0.97)	0.033
Data lacking	16			
River close				
No	197	13 (6.6)	1	
Yes	69	10 (14.5)	2.2 (1.01–4.78)	0.045
Data lacking	13			
Water source				
Pipe	164	10 (6.1)	1	
Pump, well, or borehole	88	10 (11.4)	1.86 (0.81–4.31)	0.140
Other	14	2 (14.3)	2.34 (0.57–9.66)	0.241
Data lacking	13			

*PARV4, parvovirus 4; RR, relative risk; CI, confidence interval. †Other socioeconomic factors recorded, all without association with PARV4 genotype 3 viremia, were occupation of mother, education of mother, occupation of father, education of father, number of pregnancies, age of mother, age of father, no. children in household, no. adults in household, house type, no. rooms in house, financial situation, knowledge of malaria protection, mosquito protection, electricity in household, availability of radio or television. ‡Socioeconomic interviews could not be conducted for 11 participants, and 5 questionnaires were incomplete. §χ^2^ test.

Two age groups were randomly selected from the cohort: 1) 94 infants with a median age of 14.9 months (interquartile range 14.5–15.5 months; 47 male, 47 female) and 2) 185 infants with a median age of 24.0 months (interquartile range 23.5–24.2 months; 92 male, 93 female). Significantly more positive results were seen among infants in the older versus the younger age group (22/185 [11.9%] vs. 2/94 [2.1%], respectively; p<0.006). Viral loads in the whole study group ranged from 420 to 56,000 copies/mL of whole blood (median 3,400 copies/mL). Median viral loads did not differ significantly between the age groups (4,300 vs. 3,200 copies/mL).

A 746-nt fragment of open reading frame (ORF) 1 and a 558-nt fragment of the ORF2 gene and the noncoding region between them were sequenced. Maximal nucleotide distances from previously known PARV4 sequences were 7%–8%. To obtain highly informative sequence datasets for phylogenetic analysis, we concatenated ORF1 and 2 sequences and excluded interfragment recombination by using SimPlot and GARD analysis with the HYPHY package (*13*). We then conducted phylogenetic analysis on the concatenated fragments by using the neighbor-joining method (Figure). The novel viruses from Ghana clustered in a monophyletic clade with the Nigerian PARV4 genotype 3 prototype strain NG-OR. Within this clade, sequences 1, 3, 4, 10, 12, 13, 17, 18, 19, 20, 21, 23, and 24, sampled from the 2 neighboring villages Asamang and Kona, formed a separate monophyletic subclade, suggesting local epidemic transmission of a unique virus lineage.

**Figure:**
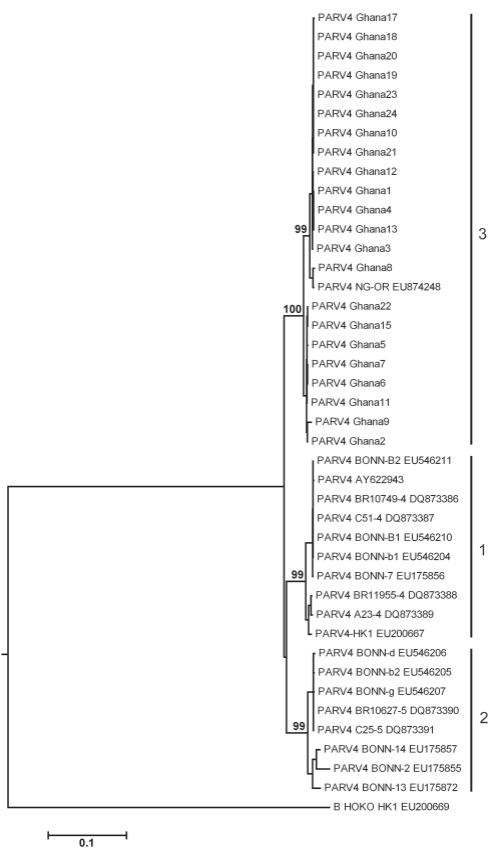
Phylogenetic analysis of human parvovirus 4 (PARV4) nucleotide sequences. The concatenated dataset of 746 open reading frame (ORF) 1 nt and 558 ORF2 nt (except for strains 14 [550 nt sequenced in ORF1] and 16 [218 nt sequenced in ORF2]) was subjected to neighbor-joining–based phylogenetic analysis with 1,000 bootstrap replicates in MEGA4 by using the Kimura substitution model and the complete deletion option for gaps (*14*). Bovine hokovirus was used as an outgroup because it is the closest relative to human PARV4. Numbers next to branches indicate bootstrap support values in percent (only selected branches are annotated). Numbers next to strain designations indicate PARV4 genotypes 1, 2, and 3. Sequences are deposited in GenBank (accession nos. GU951546–GU951569); sequencing primers available upon request from the authors. Scale bar indicates nucleotide substitutions per site.

## Conclusions

Although infection with human parvovirus PARV4 has been considered to be restricted to adults and transmitted parenterally, we found high prevalence of PARV4 genotype 3 in blood of infants in Africa. In agreement with findings for the 2 adults with PARV4 genotype 3 infection (*9*), clinical signs in these children were not overt. PARV4 infection might thus be clinically silent, or acute infection might be followed by low-level viremia that is cleared slowly, similar to infection with B19.

Finding PARV4 genotype 3 over a 2-year period in several villages suggests common and ubiquitous prevalence. Parenteral medical treatment could be clearly ruled out as a transmission route for the viremic children >2 months of age because these children were under medical observation. It could not be ruled out for younger children; however, because such treatment is uncommon in the rural Ashanti region, it is unlikely. The possibility of virus transmission by vaccination with inadequately sterilized needles is excluded because vaccination was conducted with single-used syringes. Finding virus in persons without parenteral exposure overlaps with a recently raised suspicion regarding a different epidemiology of PARV4 infection in Africa as opposed to the Northern Hemisphere (*9*).

High prevalence in children in Africa contrasts with the prevalence pattern in the Northern Hemisphere, suggesting different dynamics and routes of transmission. Prenatal or perinatal transient infection can be largely ruled out because the older children had substantially higher rates of viremia. On the contrary, our analysis of socioeconomic factors identified a reciprocal association of transmission risk with access to a kitchen and distance from a river. Foodborne or smear transmission (contact with contaminated objects) are thus suspected. In conclusion, in Africa, novel PARV4 genotype 3 is prevalent among infants who are most likely not at risk for parenteral exposure.
